# Risk factors for 90-day unplanned readmission after open posterior lumbar fusion in the elderly

**DOI:** 10.3389/fneur.2025.1653957

**Published:** 2026-01-13

**Authors:** Hao-Zhen Lyu, Lang Hu

**Affiliations:** 1 Department of Spine Surgery, Yantai Yuhuangding Hospital of Qingdao University, Yantai, Shandong, China; 2 Department of Orthopedics, Xiangyang No.1 People's Hospital, Hubei University of Medicine, Xiangyang, Hubei, China

**Keywords:** elderly, lumbar degenerative diseases, open posterior lumbar fusion, readmission within 90 days, risk factors

## Abstract

**Background:**

The inescapable trend of an aging population has made lumbar fusion increasingly common in elderly individuals. This study aimed to explore the risk factors associated with unplanned readmission within 90 days after open posterior lumbar fusion (OPLF) in elderly patients (≥ 60 years old).

**Methods:**

We retrospectively analyzed clinical data of patients who underwent OPLF at two spine centers between June 2010 and June 2024. Patients were divided into readmission and non-readmission groups according to whether they were unplanned readmitted within 90 days of the primary surgery. Demographic and clinical outcomes were compared between the two groups. Multivariate logistic regression was used to analyze risk factors for 90-day readmission.

**Results:**

Of the total, 8.6% (157/1826) of elderly patients experienced unplanned readmission within 90 days of the initial surgery. Factors including age, body mass index (BMI), American Society of Anesthesiologists (ASA) score (grade 3), history of diabetes, heart disease, respiratory disease, preoperative malnutrition, severe osteoporosis (T < −3.5), incidental durotomy, surgical segment, and surgical time in the readmission group were significantly higher than those in the non-readmission group. Multivariate logistic regression analysis suggested that higher age (*p* = 0.040, OR: 1.040, 95% CI: 1.002–1.079), ASA score ≥ grade 3 (*p* = 0.022, OR: 1.634, 95% CI: 1.074–2.485), heart disease (*p* = 0.021, OR: 1.971, 95% CI: 1.107–3.511), preoperative malnutrition (*p* = 0.028, OR: 1.701, 95% CI: 1.058–2.734), severe osteoporosis (*p* = 0.029, OR: 1.652, 95% CI: 1.054–2.588), surgical segment (*p* = 0.020, OR: 1.521, 95% CI: 1.067–2.169), and incidental durotomy (*p* = 0.012, OR: 2.193, 95% CI: 1.189–4.045) were risk factors for unplanned readmission.

**Conclusion:**

We identified seven risk factors associated with unplanned readmission within 90 days after OPLF in elderly patients. This information may assist clinicians in preoperative evaluations of patients to develop better surgical strategies.

## Introduction

Population aging is a global trend (1, 2). Reports show that the average age increased from 26.6 years in 1950 to 32.1 years in 2017 (1). Moreover, global life expectancy is projected to increase by 4.4 years for men and 4.4 years for women by 2040. In Japan, Singapore, Spain, and Switzerland, the life expectancy is over 85 years for both men and women, and in 59 countries, including China, life expectancy is projected to exceed 80 years by 2040 (2). Consequently, diseases associated with aging, such as lumbar degenerative diseases (LDD) including lumbar spinal stenosis and degenerative lumbar spondylolisthesis, are becoming increasingly common in the clinic (3). Reports show that 266 million people (3.63%) worldwide suffer from LDD and lower-back pain annually. With the development of spinal surgery technology, an increasing number of elderly individuals are undergoing lumbar fusion surgery to improve the symptoms of LDD (4–6). However, this surgery is associated with many procedure-related complications and leads to higher readmission rates relative to those of younger patients (7–9).

Reasons for readmission after spinal surgery include surgery-related factors such as incision complications, postoperative recurrence, poor improvement of patient symptoms (such as lower-back and leg pain), and other non-surgery-related factors such as respiratory, cardiac, or brain diseases (10).

Several previous studies examined the risk factors for 30-day and 90-day hospital readmission in patients undergoing spinal surgery (7–38). However, considerable heterogeneity of risk factors was associated with the types of cases (spinal tumor, fracture, degeneration, deformity) (8, 32), types of surgeries (decompression or fusion surgery, minimally invasive or open surgery) (21, 32, 36), surgical approaches (anterior or posterior) (16, 23, 28, 29, 31, 33), and surgical sites (cervical, thoracic, or lumbar spine) (33). Moreover, after spinal fusion, older patients were more likely than younger patients to be readmitted. Notably, few studies have examined the risk of early hospital readmission after lumbar fusion in elderly patients (10, 26). However, unlike the generally good postoperative status of younger patients, elderly patients may have a higher rate of unplanned readmission after surgery and more postoperative complications. Based on previous studies, this study aims to explore risk factors of 90-day unplanned readmission after open posterior lumbar fusion (OPLF) surgery in elderly patients (≥ 60 years) to provide better preoperative evaluation and surgical strategies.

## Materials and methods

We retrospectively analyzed the clinical data of patients who underwent OPLF at two spine centers between June 2010 and June 2024. The hospital ethics committee reviewed and approved the study. Due to the retrospective nature of this study, the informed consent was waived for all patients.

### Inclusion criteria

(1) Patients aged ≥ 60 years; (2) patients with LDD who underwent posterior lumbar fusion (PLF) surgery; and (3) patients with at least 3 months of follow-up.

### Exclusion criteria

(1) Patients aged < 60 years (2); patients with lumbar fractures, infections, and tumors; (3) patients with a follow-up duration of < 3 months; (4) patients undergoing anterior or lateral lumbar fusion surgery; (5) patients with tumors associated with other systems; and (6) patients readmitted > 90 days after surgery.

### Patient screening

Based on the hospital case system, we screened patients who underwent lumbar fusion surgery and included or excluded patients based on the criteria described above. In this study, elderly patients were defined as those aged 60 years or older.

### Collection of patient information

An independent investigator recorded clinical information of the enrolled patients. The information included patient sex, age, body mass index (BMI), diagnosis, smoking status, comorbidities (diabetes, hypertension, heart diseases, respiratory diseases, gastrointestinal diseases, kidney and urinary diseases, and rheumatoid arthritis), preoperative malnutrition, preoperative anemia, osteoporosis, American Society of Anesthesiologists (ASA) score, surgical method, surgical segment, surgical time, and incidental durotomy. Preoperative malnutrition was defined as a preoperative serum albumin level < 35 g/L. Diabetes and hypertension were defined according to the international norms. Brain diseases include cerebral infarction, cerebral hemorrhage, degenerative brain disease, and cerebrovascular disease. Heart diseases included cardiac insufficiency, chronic heart failure, arrhythmia, coronary heart disease, and myocardial infarction. Respiratory diseases included pneumonia, chronic bronchitis, chronic obstructive pulmonary disease, and bronchiectasis. Gastrointestinal diseases included chronic gastroenteritis, gastrointestinal ulcers, hepatitis, cholecystitis with gallstones and cholangitis with bile duct stones. Kidney and urinary diseases included chronic nephritis, chronic renal failure, uremia, prostate enlargement, and kidney, ureter, and bladder stones. Anemia was defined as hemoglobin levels < 110 g/L in women and < 120 g/L in men. Surgical methods included posterior lumbar interbody fusion (PLIF), transforaminal lumbar interbody fusion (TLIF), and posterolateral fusion (PLF). PLIF, TLIF, and PLF were all included in OPLF in this study. An incidental durotomy was defined as an incidental intraoperative dural tear. Osteoporosis was defined as a patient with a lumbar spine bone mineral density *T*-value of less than −2.5 (dual-energy X-ray). Severe osteoporosis was defined as a *T*-value less than −3.5.

### Grouping

The patients were divided into 90-day readmission and non-readmission groups based on readmission within 90 days after surgery. Demographic and clinical information was compared between the two groups.

### Statistical methods

A t-test was used to compare age, BMI, surgical time, and surgical segment between the two groups. Chi-square decompression or Fisher’s exact tests were used to compare sex, smoking status, comorbidities, preoperative malnutrition, preoperative anemia, osteoporosis, ASA score, surgical segment, and incidental durotomy between the two groups. Univariate logistic was used to assess the association between each variable and patient unplanned readmission 90 days after surgery. Multivariate logistic regression was used to assess risk factors for patient readmission 90 days after surgery. SPSS (version.25, IBM Corp, Armonk, NY, USA) was used for statistical analyses. *p*-values were considered statistically significant at *p* < 0.05.

## Results

Initially, 4,475 patients who underwent lumbar fusion were included in this study. After excluding 2,369 patients who were younger than 60 years of age, 33 patients with lumbar fractures, 14 patients with lumbar infections, 5 patients with lumbar tumors, 26 patients with a follow-up of less than 3 months, 138 patients who underwent anterior or lateral lumbar fusion surgery, 6 patients with other systemic tumors, and 58 patients who were readmitted more than 90 days after surgery, 1,826 patients were finally included in this study. Of these, 157 (8.6%) patients were unplanned readmitted within 90 days of the initial procedure, and 1,669 (91.4%) patients were not readmitted within 90 days.

We found no significant differences between the two groups in sex, BMI, smoking status, hypertension, brain disease, kidney and urinary diseases, gastrointestinal diseases, rheumatoid arthritis, preoperative anemia rate, or surgical methods. The values for age (69.9 ± 5.3 vs. 67.7 ± 5.0, *p* < 0.001), ASA score (42.0% vs. 24.3%, *p* < 0.001), surgical segment (1.7 ± 1.0 vs. 1.4 ± 0.7, *p* < 0.001), surgical time (173.0 ± 56.4 vs. 155.6 ± 41.5, *p* < 0.001), proportion of preoperative malnutrition (22.3% vs. 9.7%, *p* < 0.001), diabetes (13.4% vs. 6.8%, *p* = 0.002), heart disease (13.4% vs. 4.7%, *p* < 0.001), respiratory disease (12.7% vs. 5.8%, *p* < 0.001), severe osteoporosis (18.5% vs. 11.7%, *p* < 0.001), and incidental durotomy (9.6% vs. 4.3%, *p* < 0.001) in the 90-day readmission group were significantly higher than those in the non-readmission group (Table 1).

**Table 1:** Patients’ clinical parameters of the two groups.

Subgroup	Readmission group (*N* = 157)	Non- readmission group (*N* = 1,669)	*p* value
Sex			0.654
Men	78 (49.7%)	789 (47.8%)	
Women	79 (50.3%)	871 (52.2%)	
Age (year)	69.9 ± 5.3	67.7 ± 5.0	<0.001
BMI (kg/m^2^)	25.4 ± 2.6	25.2 ± 2.8	0.289
Smoking	22 (14.0%)	192 (11.5%)	0.499
Diabetes	21 (13.4%)	113 (6.8%)	0.002
Hypertension	39 (24.8%)	338 (20.3%)	0.331
Brain diseases	9 (5.7%)	55 (3.3%)	0.112
Heart diseases	21 (13.4%)	78 (4.7%)	<0.001
Respiratory diseases	20 (12.7%)	96 (5.8%)	<0.001
Gastrointestinal diseases	10 (6.4%)	73 (4.4%)	0.251
Kidney and urinary diseases	13 (8.3%)	87 (5.2%)	0.106
Rheumatoid arthritis	4 (2.5%)	153 (1.9%)	0.587
Severe osteoporosis	29 (18.5%)	196 (11.7%)	0.014
Preoperative anemia	18 (11.5%)	141 (8.4%)	0.200
Preoperative malnutrition	35 (22.3%)	162 (9.7%)	<0.001
ASA score			<0.001
Grade 1–2	91 (58.0%)	1,264 (75.7%)	
Grade 3–4	66 (42.0%)	405 (24.3%)	
Surgical segment	1.7 ± 1.0	1.4 ± 0.7	<0.001
Surgical time (mins)	173.0 ± 56.4	155.6 ± 41.5	<0.001
Surgical methods			0.557
PLIF	90 (57.3%)	885 (53.0%)	
TLIF	52 (33.1%)	594 (35.6%)	
PLF	15 (9.6%)	190 (12.1%)	
Incidental durotomy	15 (9.6%)	71 (4.3%)	0.003

BMI, body mass index; ASA, American Society of Anesthesiologists; PLIF, Posterior lumbar interbody fusion; TLIF, Transforaminal lumbar interbody fusion; PLF, Posterolateral fusion.

Univariate logistic regression analysis showed that age (*p* < 0.001, OR: 1.083, 95% CI: 1.050–1.117), ASA score (*p* < 0.001, OR: 2.264, 95% CI: 1.617–3.168), surgical segment (*p* < 0.001, OR: 1.579, 95% CI: 1.344–1.855), surgical time (*p* < 0.001, OR: 1.008, 95% CI: 1.005–1.011), preoperative malnutrition (*p* < 0.001, OR: 2.669, 95% CI: 1.772–4.018), diabetes mellitus (*p* < 0.001, OR: 2.126, 95% CI: 1.293–3.497), heart disease (*p* < 0.001, OR: 3.150, 95% CI: 1.886–5.259), respiratory disease (*p* = 0.001, OR: 2.392, 95% CI: 1.443–3.993), severe osteoporosis (*p* = 0.015, OR: 1.703, 95% CI: 1.108–2.617) and incidental durotomy (*p* = 0.004, OR: 2.378, 95% CI: 1.327–4.258) were associated with 90-day unplanned readmission (Table 2).

**Table 2:** Univariate logistic regression analysis of 90 days readmission after primary surgery.

Variables	*p* value	OR	95% CI
Age	<0.001	1.083	1.050–1.117
Diabetes	0.003	2.126	1.293–3.497
Heart diseases	<0.001	3.150	1.886–5.259
Respiratory diseases	0.001	2.392	1.443–3.993
Severe osteoporosis	0.015	1.703	1.108–2.617
Preoperative malnutrition	<0.001	2.669	1.772–4.018
ASA score	<0.001	2.264	1.617–3.168
Surgical segment	<0.001	1.579	1.344–1.855
Surgical time	<0.001	1.008	1.005–1.011
Incidental durotomy	0.004	2.378	1.327–4.258

Multivariate logistic analysis showed that age (*p* = 0.040, OR: 1.040, 95% CI: 1.002–1.079), heart disease (*p* = 0.021, OR: 1.971, 95% CI: 1.107–3.511), ASA score (*p* = 0.022, OR: 1.634, 95% CI: 1.074–2.485), surgical segment (*p* = 0.020, OR: 1.521, 95% CI: 1.067–2.169), preoperative malnutrition (*p* = 0.028, OR: 1.701, 95% CI: 1.058–2.734), severe osteoporosis (*p* = 0.029, OR: 1.652, 95% CI: 1.054–2.588), and incidental durotomy (*p* = 0.012, OR: 2.193, 95% CI: 1.189–4.045) were the risk factors for 90-day unplanned readmission after PLF (Table 3). This suggests that the risk of readmission increased by approximately 4% for each year of age after adjusting for confounding factors, such as diabetes, surgical time, and chronic respiratory diseases. Patients with heart disease showed an approximately 1.971 times higher risk of readmission when compared with those who did not have heart disease. Patients who had an ASA score of 3–4 showed an approximately 1.634 times higher risk for readmission when compared with those who had an ASA score of 1–2. The risk of readmission increased by approximately 52.1% for each additional level of surgical segment. Patients with preoperative malnutrition had a risk of readmission that was approximately 1.701 times higher than that of non-malnourished patients. Patients with severe osteoporosis had a risk of readmission approximately 1.652 times higher than that of patients without severe osteoporosis. Patients who underwent incidental durotomy were readmitted approximately 2.193 times more often than those who did not.

**Table 3:** Multivariate logistic regression analysis of 90 days readmission after primary surgery.

Variables	*p* value	OR	95% CI
Age	0.040	1.040	1.002–1.079
Diabetes	0.185	1.484	0.828–2.657
Heart diseases	0.021	1.971	1.107–3.511
Chronic respiratory diseases	0.528	1.209	0.670–2.181
Severe osteoporosis	0.029	1.652	1.054–2.588
Preoperative malnutrition	0.028	1.701	1.058–2.734
ASA score	0.022	1.634	1.074–2.485
Surgical segment	0.020	1.521	1.067–2.169
Surgical time	0.752	1.001	0.994–1.008
Incidental durotomy	0.012	2.193	1.189–4.045

The factors associated with unplanned readmission within 90 days after the initial surgery included surgery-related factors (56.7%, 89/157) and non-surgery-related factors (43.3%, 68/157). Among surgery-related causes, surgical site infection was the most common (29.3%) (Table 4). Other surgery-related factors included poor wound healing (17.2%), internal fixation failure (3.8%), residual symptoms (3.2%), epidural hematoma (1.9%), and ganglionitis (1.3%). Among non-surgery-related causes, heart disease was the most common (10.2%). Other factors unrelated to surgery included hypertension (1.3%), diabetes (4.5%), brain diseases (3.8%), respiratory diseases (5.7%), gastrointestinal diseases (5.1%), kidney and urinary diseases (3.2%), rheumatoid arthritis (0.6%), additional spinal fractures (5.7%), and cervical spine surgery (3.2%).

**Table 4:** Factors for 90 days readmission after primary surgery.

Subgroup	Readmission group (*N* = 157)	Rate (%)
Surgical related factors	89	56.7
Surgical site infection	46	29.3
Poor wound healing	27	17.2
Internal fixation failure	6	3.8
Residual symptoms	5	3.2
Epidural hematoma	3	1.9
Ganglionitis	2	1.3
Non-surgical related factors	68	39.5
Hypertension	2	1.3
Diabetes	7	4.5
Brain diseases	6	3.8
Heart diseases	16	10.2
Respiratory diseases	9	5.7
Gastrointestinal diseases	5	3.2
Kidney and urinary diseases	8	5.1
Rheumatoid arthritis	1	0.6
Additional spinal fractures	9	5.7
Additional cervical spine surgeries	5	3.2

## Discussion

As the aging of the population continues to progress, more and more elderly patients are undergoing OPLF surgery. Unlike previous studies, this study specifically explored the risk factors of 90-day readmission after OPLF in elderly patients in order to better formulate a reasonable treatment strategy for the elderly patients. In this study, we found that 8.6% (157/1826) of the elderly patients experienced unplanned readmission within 90 days after the initial surgery. Moreover, we identified higher age, heart diseases, ASA score (≥ grade 3), higher surgical segment, preoperative malnutrition, severe osteoporosis and incidental durotomy as risk factors for 90-day unplanned readmission.

The effect of age on readmission in patients undergoing spinal surgery was demonstrated in several previous studies (8, 9, 16, 19, 25, 31, 32, 35). A recent retrospective study by Taliaferro et al. (8) found that age > 80 years was a risk factor for readmission to the hospital within 90 days after spinal fusion surgery. Bae et al. (9) found that when compared with young patients, elderly patients had a higher 90-day readmission rate after full-endoscopic transforaminal lumbar discectomy. Elia et al. (19) also found that the average age of the 90-day readmission group (68.58 years old) was significantly higher than that of the 90-day non-readmission group (61.76 years old) following occipitocervical fusion surgery. In this study, we found that older age was a risk factor for readmission within 90 days after OPLF. The risk of readmission increased by approximately 4% for each year of age. Older age may indicate poorer physical condition (such as anemia and malnutrition), more frequent comorbidities (such as diabetes, hypertension, heart disease, respiratory disease, etc.), and less tolerance to surgery under general anesthesia, leading to higher readmission rates soon after surgery.

Heart disease was associated with readmission after lumbar spine surgery in previous studies (15, 23, 31, 34, 35). Shah et al. (15) found that angina pectoris was a risk factor in 30-day readmission after lumbar spinal fusion. Rubel et al. (23) found that acute myocardial infarction was a risk factor for 90-day readmission after elective primary lumbar spine surgery. A recent systematic review by Chen et al. (31) found that heart failure was associated with readmission within 30 days after surgery for LDD. In the present study, we found that cardiac disease was a risk factor for hospital readmission. Patients with heart disease had an approximately 1.971 times higher risk of readmission than those who did not have heart disease. These findings underscore that surgeons should fully inform patients and not neglect management of heart disease to avoid readmission after surgery.

Higher ASA scores were associated with higher readmission rates in several previous studies (7, 11, 16, 21, 22, 28, 31, 32). Kim et al. (7) found that an ASA score of > 2 could predict 30-day readmission after cervical laminoplasty. Wadhwa et al. (11) found that a higher ASA score was associated with 90-day readmission following lumbar spinal surgery. These studies found that in general, an ASA score ≥ 3 was a risk factor for 30-day or 90-day readmission after spinal surgery. In this study, we found that an ASA score ≥ 3 was a risk factor for readmission after OPLF. Patients with an ASA score of 3–4 had an approximately 1.634 times higher risk of readmission than those with an ASA score of 1–2. This suggests to surgeons that more aggressive preoperative conditioning may be necessary in patients with ASA scores ≥ 3.

Longer surgical segments are also associated with early admission after spinal surgery (8, 29, 31, 33). In this study, the surgical segments and durations in the readmission group were significantly higher than those in the non-readmission group. Multivariate logistic analysis suggested that surgical segment, rather than surgical time, was a risk factor for readmission. The risk of readmission increased by approximately 52.1% for each additional level of surgical segment. This finding could be explained by longer surgical segments resulting in longer surgical times, higher blood loss, trauma, and a higher incidence of complications. The interactions between these factors led to an increase in early readmission rates.

Studies showed that the nutritional status of patients affected the results of spinal surgery during the perioperative period (39–41). A recent propensity score study by Elsamadicy et al. (39) found that malnourished patients who underwent lumbar fusion for spondylolisthesis had significantly higher rates of adverse events, unplanned readmissions, and longer-term lengths of stay than nourished patients. In this study, we also found that patients with preoperative malnutrition had a risk of readmission that was approximately 1.701 times higher than that of non-malnourished patients. This suggests that correcting malnutrition in elderly patients is crucial before undergoing OPLF surgery.

Previous reports showed that osteoporosis affected the clinical outcomes of lumbar fusion surgery (18, 42, 43). A recent study by Lee et al. (18) found that osteoporosis was significantly associated with higher readmission rates, longer hospital stays, and higher medical costs after spinal surgery. Jain et al. (42) also found that osteoporosis was a risk factor for 30-day readmission after 1–2-level elective posterior lumbar fusions. In our study, severe osteoporosis was significantly associated with 90-day readmission. The risk of readmission in patients with severe osteoporosis was approximately 1.652 times higher than that of patients without severe osteoporosis. In addition, of 157 readmitted patients, 6 (3.8%) had failed internal fixation. These results are a reminder to surgeons of the importance of standardized anti-osteoporosis measures after internal fixation and fusion surgery because severe osteoporosis is a risk factor for screw loosening, proximal junctional kyphosis, and revision surgery.

Incidental durotomy has also been associated with early readmission following spinal surgery in previous studies (30, 44). Rumalla et al. (30) found that incidental durotomy was a risk factor for 30-day readmission after degenerative posterior cervical spine surgery. Kohls et al. (44) found that incidental durotomy was related to 90-day readmission after lumbar discectomy. In this study, we found that patients who underwent incidental durotomy were approximately 2.193 times more likely to be readmitted than those who did not. The main causes included poor wound healing or surgical-site infections caused by incidental durotomy. Consequently, meticulous surgery should be performed on elderly patients to avoid incidental durotomy.

As reported by Lee et al. (10), in elderly patients (> 70-years-old), the reason for readmission within 360 days after degenerative lumbar spine surgery is often more related to non-surgical-site issues rather than those directly related to surgery. In this study, we found that 56.7% (89/159) of readmissions were due to surgery-related factors vs. 43.3% (68/157) to non-surgery-related factors.

These results strongly suggest that surgeons should consider more factors to reduce unplanned readmission rates in elderly patients.

### Limitations

First, the retrospective nature of this study might have led to unavoidable biases, such as the selection of cases and surgical methods. Second, although the patients in this study were from two spine centers, the overall volume of data in the study is still limited. Additionally, this retrospective design spanning 14 years (2010–2024) may ignore the substantial evolution in surgical techniques, perioperative care protocols, and patient selection criteria over this period, which may potentially confounding results; temporal trends were acknowledged but not adjusted for. Surgical techniques were not stratified; although PLIF, TLIF, and PLF were grouped under OPLF, outcomes may differ by approach, possibly obscuring specific risk patterns. Additionally, both centers are in China, which may limit generalizability to healthcare systems with different perioperative protocols and discharge practices. Finally, the study did not include all factors, such as some very rare comorbidities including immunodeficiency, thrombocytopenia, Sjogren’s syndrome, uterine prolapse, varicose veins, sarcopenia, pre-operative paraspinal muscle status, etc., as these comorbidities only exist in a very small number of patients. Additionally, the socioeconomic factors such as the patient’s medical insurance type and marital status were not included in this study. Thus, we concluded that these factors should not be considered.

## Conclusion

We found that seven risk factors were associated with unplanned readmission within 90 days in elderly patients after undergoing OPLF. These factors included older age, heart diseases, ASA scores ≥ grade 3, higher surgical segments, preoperative malnutrition, severe osteoporosis, and incidental durotomy. This information may assist clinicians in performing better preoperative evaluations resulting in better surgical strategies.

## Data Availability

The original contributions presented in the study are included in the article/supplementary material, further inquiries can be directed to the corresponding author.
